# Small Molecule Anti-biofilm Agents Developed on the Basis of Mechanistic Understanding of Biofilm Formation

**DOI:** 10.3389/fchem.2019.00742

**Published:** 2019-11-01

**Authors:** Katrine Qvortrup, Louise Dahl Hultqvist, Martin Nilsson, Tim Holm Jakobsen, Charlotte Uldahl Jansen, Jesper Uhd, Jens Bo Andersen, Thomas E. Nielsen, Michael Givskov, Tim Tolker-Nielsen

**Affiliations:** ^1^Department of Chemistry, Technical University of Denmark, Lyngby, Denmark; ^2^Department of Immunology and Microbiology, Costerton Biofilm Center, Faculty of Health and Medical Sciences, University of Copenhagen, Copenhagen, Denmark; ^3^Singapore Centre for Environmental Life Sciences Engineering, Nanyang Technological University, Singapore, Singapore

**Keywords:** *Pseudomonas aeruginosa*, *Escherichia coli*, *Acinetobacter baumannii*, c-di-GMP, pilicides and curlicides, quorum sensing, anti-biofilm compounds

## Abstract

Microbial biofilms are the cause of persistent infections associated with various medical implants and distinct body sites such as the urinary tract, lungs, and wounds. Compared with their free living counterparts, bacteria in biofilms display a highly increased resistance to immune system activities and antibiotic treatment. Therefore, biofilm infections are difficult or impossible to treat with our current armory of antibiotics. The challenges associated with biofilm infections have urged researchers to pursue a better understanding of the molecular mechanisms that are involved in the formation and dispersal of biofilms, and this has led to the identification of several steps that could be targeted in order to eradicate these challenging infections. Here we describe mechanisms that are involved in the regulation of biofilm development in *Pseudomonas aeruginosa, Escherichia coli*, and *Acinetobacter baumannii*, and provide examples of chemical compounds that have been developed to specifically inhibit these processes. These compounds include (i) pilicides and curlicides which inhibit the initial steps of biofilm formation by *E. coli*; (ii) compounds that interfere with c-di-GMP signaling in *P. aeruginosa* and *E. coli*; and (iii) compounds that inhibit quorum-sensing in *P. aeruginosa* and *A. baumannii*. In cases where compound series have a defined molecular target, we focus on elucidating structure activity relationship (SAR) trends within the particular compound series.

## Introduction

During the last two decades it has been realized that the biofilm mode of growth is the predominant life-mode of most bacterial species (Flemming and Wuertz, 2019). In biofilms bacteria are located in densely packed microcolonies concealed in a protective matrix of biopolymers (Tolker-Nielsen, 2015). The ability of bacteria to form biofilms is an ancient trait that during evolution has offered protection from grazing amoebae and antimicrobials. When higher organisms evolved, bacteria adapted to form biofilms at various body sites, and today the extensive use of medical devises in modern health care has provided numerous new niches for bacterial biofilm formation. In the biofilm life-mode the bacteria are tolerant to our present assortment of antibiotics as well as immune system activities (Rybtke et al., 2015; Ciofu and Tolker-Nielsen, 2019). Accordingly, biofilms are the cause of persistent infections associated with various medical implants, and distinct disease states such as urinary tract infection, cystic fibrosis, chronic obstructive pulmonary disease and chronic wounds. The US National Institutes of Health has estimated that more than 80% of all microbial infections in the developed countries involve biofilms.

The problematic infections caused by biofilms have urged researchers to study the molecular mechanisms underlying biofilm formation and biofilm dispersal. This has led to the identification of distinct molecules and mechanisms that could serve as target for novel anti-biofilm drugs. Based on this knowledge, development of several potential anti-biofilm drug candidates is underway. Here we describe our current knowledge of the molecular mechanisms involved in biofilm development for the Gram negative opportunistic pathogens *Pseudomonas aeruginosa, Escherichia coli*, and *Acinetobacter baumannii*, which are all causing problematic biofilm infections. Subsequently, we provide examples of chemical compounds that have been developed to inhibit specific biofilm formation processes. These compounds include i) pilicides and curlicides which inhibit the initial steps of biofilm formation by *E. coli*; ii) compounds that interfere with c-di-GMP signaling in *P. aeruginosa* and *E. coli*; and iii) compounds that inhibit quorum sensing in *P. aeruginosa* and *A. baumannii*.

## Molecular Mechanisms Involved in Biofilm Formation

In this section we review biofilm formation processes of *P. aeruginosa, E. coli*, and *A. baumannii* with an emphasis on the molecular mechanisms which are used as targets for the development of anti-biofilm chemicals.

Biofilm formation requires an extracellular matrix that under most conditions is produced by the biofilm forming organism. Compelling evidence suggests that various bacterial species employ so-called c-di-GMP signaling to regulate whether they produce an extracellular matrix and form biofilm, or assume a planktonic lifestyle (Fazli et al., 2014; Jenal et al., 2017). Diguanylate cyclase enzymes (DGCs) catalyze formation of the molecule c-di-GMP, whereas specific phosphodiesterase enzymes (PDEs) catalyze degradation of c-di-GMP in the bacteria. Bacteria typically produce several different DGCs and PDEs, and the available evidence suggests that specificity of c-di-GMP signaling is warranted through physical interactions of specific DGC, PDE, and c-di-GMP effectors (Sarenko et al., 2017). In addition to their catalytic domains these enzymes often contain regulatory domains, and are thought to regulate the life-style (planktonic vs. biofilm) of bacteria in response to environmental cues. An elevated cellular level of c-di-GMP induces the production of biofilm matrix components and drives bacteria to form biofilms, whereas a reduction in the c-di-GMP level down regulates the production of biofilm matrix components and causes dispersal of biofilm bacteria into the planktonic mode of life (Gjermansen et al., 2006, 2010; Christensen et al., 2013). The DGCs and PDEs that make and break c-di-GMP have conserved catalytic GGDEF (Gly-Gly-Asp-Glu-Phe) and EAL (Glu-Ala-Leu)/HD-GYP (His-Asp—Gly-Tyr-Pro) domains, respectively. Genomic analyses have shown that the GGDEF and EAL domains are the most abundant motives among *Bacteria*. Accordingly, c-di-GMP has been found to be the central biofilm regulator in all Gram negative bacteria investigated to date, and in a number of Gram positive bacteria (Jenal et al., 2017). On the contrary, c-di-GMP is not produced by humans or other mammals.

Quorum sensing (QS) is another regulatory process that plays a role in biofilm formation of a variety of bacterial species, and also functions as an overall regulator of the expression of virulence factors (Juhas et al., 2005). QS is a bacterial cell-to-cell communication process that relies on the production, sensing and response to extracellular signaling molecules. QS allows groups of bacteria to synchronously alter the expression of specific genes, mainly in response to changes in their population density. In Gram negative bacteria the majority of QS systems employ acyl homoserine lactone (AHL) signal molecules. The systems function by means of one or more AHL synthases that produce AHL molecules which can pass the bacterial membranes and upon reaching a threshold concentration binds to and activates one or more transcriptional activators which in turn activate transcription of specific target genes. The concentrations of signal molecules correlate to the density of the bacterial population, enabling density dependent control of gene expression.

### Pseudomonas aeruginosa

*P. aeruginosa* biofilms are causing a number of persistent infections, including cystic fibrosis pneumonia, chronic obstructive pulmonary disease related infections, chronic wound infections, chronic otitis media, chronic bacterial prostatitis, and medical device-related infections especially associated with urinary tract catheters and endotracheal tubes (Tolker-Nielsen, 2014).

Biofilm formation by *P. aeruginosa* can initiate through the adhesive action of several components, including flagella (O'Toole and Kolter, 1998), type IV pili (O'Toole and Kolter, 1998; Deziel et al., 2001; Chiang and Burrows, 2003), Cup fimbria (Vallet et al., 2001), extracellular DNA (Whitchurch et al., 2002), and Psl polysaccharide (Ma et al., 2006). Many of these components are also important constituents of the extracellular matrix at later stages of biofilm formation. The exopolysaccharide Psl is cell-surface associated and functions as an adhesin in the initial phase of biofilm formation, but relocates as a peripheral exopolysaccharide at later stages of biofilm formation (Ma et al., 2009). The surface adhesin CdrA binds to Psl, and it is required for Psl-mediated aggregation of *P. aeruginosa* cells (Borlee et al., 2010). Pel is a cationic exopolysaccharide produced by *P aeruginosa*, and it has been shown to cross-link extracellular DNA in the biofilm matrix (Jennings et al., 2015). Overproduction of alginate exopolysaccharide enables mucoid *P. aeruginosa* strains to form biofilm persistent infections in the lungs of cystic fibrosis patients (Hoiby, 1977). Moreover, *P. aeruginosa* rugose small colony variants that overproduce Psl and Pel exopolysaccharide show enhanced persistence in cystic fibrosis lungs (Starkey et al., 2009). In addition, the lectins LecA/LecB and the functional amyloid protein Fap can play a role as matrix components in *P. aeruginosa* biofilms (Tielker et al., 2005; Diggle et al., 2006b; Dueholm et al., 2013).

Synthesis in *P. aeruginosa* of the biofilm matrix components Psl, Pel, alginate, CdrA, type IV pili, and Cup fimbriae is positively regulated by c-di-GMP (Fazli et al., 2014). Conversely, c-di-GMP is a negative regulator of motility of *P. aeruginosa* (Simm et al., 2004). The c-di-GMP content in *P. aeruginosa* is adjusted via the activity of 17 proteins with a GGDEF domain, 9 proteins with an EAL/HD-GYP domain, and 16 proteins with both a GGDEF and an EAL domain. Christensen et al. (2013) constructed a recombinant bacterial *P. aeruginosa* strain, where the cellular level of c-di-GMP can be reduced by addition of an inducer that activates transcription of a PDE gene, and demonstrated that *in vitro P. aeruginosa* biofilms disperse in response to a reduction in the cellular c-di-GMP content (Christensen et al., 2013). In addition, evidence was provided that murine implant-associated *P. aeruginosa* biofilm infections can be cured through a reduction of the bacterial c-di-GMP content (Christensen et al., 2013).

QS in *P. aeruginosa* is mediated through the LasI/LasR and RhlI/RhlR proteins that produces and senses the signal molecules 3-oxo-C12-homoserine lactone (HSL) and C4-homoserine lactone (C4-HSL) (Schuster and Greenberg, 2006). Moreover, *P. aeruginosa* employs a Pqs system that produces and senses 2-heptyl-3-hydroxy-4-quinolone (termed PQS) (Diggle et al., 2006a). The systems are hierarchically arranged with LasR regulating the Rhl and Pqs systems (Diggle et al., 2006a). QS regulates the production of a number of compounds that play a role in the formation and persistence of *P. aeruginosa* biofilms. Among these QS-regulated factors is extracellular DNA that contributes to the stability of *P. aeruginosa* biofilms and plays a role in the antimicrobial tolerance displayed by the biofilms (Allesen-Holm et al., 2006; Chiang et al., 2013). Moreover, the production of rhamnolipid is QS regulated, and this amphipathic molecule plays a role in biofilm development and resistance of *P. aeruginosa* biofilms to phagocytizing immune cells (Pamp and Tolker-Nielsen, 2007; Alhede et al., 2009). Thus, inhibition of QS decreases the antibiotic tolerance of *P. aeruginosa* biofilms and reduces resistance to host immune responses (Jakobsen et al., 2017a).

### Escherichia coli

*E. coli* is the primary infectious agent in urinary tract infections, including cystitis (infection of the bladder or lower part of the urinary tract) and pyelonephritis (infection of the kidneys or infections associated with the upper urinary tract). The bacterium is able to form biofilms on catheter material, on the bladder wall, and within bladder epithelial cells, and biofilm formation is often related to infection relapses (Anderson et al., 2003; Justice et al., 2004; Soto et al., 2006; Rosen et al., 2007, 2008).

Biofilm formation by *E. coli* can be governed by a number of different adhesins and extracellular matrix components. Flagella may play a role in transport of the bacteria to a surface, and in adhesion to the surface (Pratt and Kolter, 1998). The proteinaceous curli fibers are a major component of the *E. coli* biofilm matrix, and they are required in the initial stages of *E. coli* attachment to proteins on the host cells (Olsen et al., 1989; Ben Nasr et al., 1996; Prigent-Combaret et al., 2000; Chapman et al., 2002; Serra et al., 2013). Curli fibers are comprised of the two proteins, CsgA and CsgB, where CsgB primes the polymerization of CsgA (Hammar et al., 1995). Type 1 and P pili are adhesive surface structures which are important in *E. coli* infections (Pratt and Kolter, 1998; Schembri and Klemm, 2001; Niba et al., 2007). Type 1 pili are central for the irreversible attachment of the cells to a surface, and type 1 pili deficient strains shows significant impairment in biofilm formation (Pratt and Kolter, 1998; Niba et al., 2007). Using a mouse model, it has been shown that uropathogenic *E. coli* (UPEC) strains, that expresses type 1 pili, have a survival advantage in the bladder (Mulvey et al., 1998), and that *E. coli* lacking type 1 or P pili is greatly attenuated in causing urinary tract infections (Sivick and Mobley, 2010). Type 1 pili contain repeating FimA subunits which assemble to a coli-rod structure that is tipped with FimH adhesin molecules (Brinton, 1965; Barnhart et al., 2003; Korea et al., 2011). Absence of FimH reduces adhesion both *in vitro* and in the bladders of mice (Langermann et al., 1997; Mulvey et al., 1998). FimH is responsible for a specific adhesion to mannose residues on epithelial cells, thereby facilitating infection (Pratt and Kolter, 1998; Hertig and Vogel, 2012; Rakshit and Sivasankar, 2014). P pili contain PapA subunits assembled to a helical structure, which at the tip anchors the adhesin PapG (Gong and Makowski, 1992; Bullitt and Makowski, 1995). PapG enables *E. coli* cells to bind to epithelial kidney cells in the host (Busch et al., 2015). Antigen43 is an autotransporter protein, which has been linked with higher intracellular persistence in urinary tract infections (Anderson et al., 2003; Klemm and Schembri, 2004; Van Der Woude and Henderson, 2008; Luthje and Brauner, 2010). In addition to the protein components, the exopolysaccharides cellulose, poly-GlcNAc (PGA), and colanic acid are important structural components of the matrix of *E. coli* biofilms (Danese et al., 2000; Wang et al., 2004; Serra et al., 2013; Subashchandrabose et al., 2013; Besharova et al., 2016). Moreover, extracellular DNA can function as an important matrix component in *E. coli* biofilms (Devaraj et al., 2015).

Synthesis in *E. coli* of the biofilm matrix components cellulose, PGA and curli fimbria is positively regulated by c-di-GMP (Brown et al., 2001; Brombacher et al., 2003, 2006; Weber et al., 2006; Jonas et al., 2008; Pesavento et al., 2008; Boehm et al., 2009). Conversely, c-di-GMP is a negative regulator of motility of *E. coli* (Simm et al., 2004). The c-di-GMP content in *E. coli* is determined by the activity of several GGDEF/EAL/HD-GYP domain proteins; e.g., the lab strain *E. coli* K12 possess 12 DGCs and 13 PDEs (Hengge et al., 2016; Povolotsky and Hengge, 2016). Evidence has been provided that there is a hierarchical arrangement of DGCs and PDEs, with a few master controllers (Sarenko et al., 2017). The PDE, PdeH (formerly YhjH) was shown to eradicate global effects of several DGCs, thereby limiting them to act on local systems. The major DGC was found to be DgcE, which is a primary controller of the c-di-GMP level in *E. coli* (Sarenko et al., 2017).

*E. coli* does not encode an AHL based QS system. However, it evidently employs a furanosyl borate diester (AI-2) based QS system, and evidence has been presented that this system is involved in regulation of the synthesis of some of the biofilm matrix components (Beloin et al., 2008).

### Acinetobacter baumannii

The most common infections caused by *A. baumannii* are pneumonia, meningitis, urinary tract infection, skin and soft tissue infection, wound infections and bacteremia (Visca et al., 2011). The increased use of mechanical ventilation and central venous and urinary catherization has greatly increased the incidence of *A. baumanni* infections (Wong et al., 2017). The ability of *A. baumanni* to form biofilm is contributing significantly to the recalcitrance of these infections to antibiotic treatment.

The mechanisms involved in biofilm formation for *A. baumanni* are less studied than for *P. aeruginosa* and *E. coli*. However, a number of adhesins and extracellular components have been identified as playing a role in biofilm formation of *A. baumanni*. The bacteria lack flagella, but surface adhesion is facilitated by Csu pili and the OmpA outer membrane protein (Dorsey et al., 2002; Tomaras et al., 2003; Gaddy et al., 2009). Besides adhesion to abiotic surfaces, OmpA binds to epithelial cells and thus mediate biofilm formation on biotic surfaces (Gaddy et al., 2009). In addition, a biofilm associated protein (Bap) has been reported to play a role in *A. baumanni* biofilm formation (Loehfelm et al., 2008). Evidence was provided that Bap is important in cell-cell adhesion and in keeping the structure of mature biofilms (Loehfelm et al., 2008). Moreover, *A. baumanni* can synthesize the exopolysaccharides alginate and poly-β-1,6-N-acetylglucosamine (PNAG) which can function as important constituents of the biofilm matrix (Lee et al., 2008; Choi et al., 2009). Extracellular DNA is also a matrix component in *A. baumannii* biofilms (Sahu et al., 2012).

There is a lack of knowledge regarding a role of c-di-GMP signaling in the regulation of biofilm formation by *A. baumannii*. However, a study employing an *in silico* pharmacophore-based screen to identify small-molecule inhibitors of DGC enzymes, found that the hit compounds could inhibit biofilm formation of *A. baumannii* (Sambanthamoorthy et al., 2014). Synthesis of Csu pili is known to be regulated by the BfmSR two-component system in *A. baumannii* (Tomaras et al., 2008; Liou et al., 2014).

QS has been shown to play a role in regulation of biofilm formation by *Acinetobacter* species (Anbazhagan et al., 2012). *A. baumannii* possess an AHL-based QS system with AbaI functioning as the AHL synthase and AbaR functioning as the AHL receptor. An *abaI* mutant, not able to produce AHL, was shown to have defects in the later stages of biofilm formation (Niu et al., 2008). In addition, it was shown that addition of AHL to *A. baumannii* cells resulted in an increased expression of Csu pili, and a stimulation of biofilm formation (Luo et al., 2015). Moreover, a low concentration of Fe^3+^ was found to induce AHL-based QS in *A. baumannii*, and to promote the formation of robust biofilms (Modarresi et al., 2015). *A. baumannii* is capable of producing a quorum quenching enzyme designated AidA, and Lopes at al. found that activation of AidA in *A. baumannii* resulted in inhibition of biofilm formation (Lopez et al., 2017).

## Compounds That Interfere With Biofilm Formation

In this section we describe pilicides and curlicides which inhibit the initial steps of biofilm formation by *E. coli*, and compounds that interfere with c-di-GMP signaling in *P. aeruginosa* and *E. coli*, as well as compounds that inhibit QS in *P. aeruginosa* and *A. baumannii*. A direct comparison of compound efficiencies is difficult due to the lack of standardization between assays used in the different studies. Even in studies investigating modulation of the same protein, there is often a variation in the used bacterial strains, growth media and assay. This makes a quantitative comparison of activities (e.g., IC50 values) from various studies problematic and in some cases even misleading. Therefore, this review is focused on reviewing activity trends observed within each assay as well as giving a more qualitative comparison of structures and relative biological effects across different studies.

### Compounds That Modulate the Function of Pili and Curli in *Escherichia coli*

Bacterial attachment is an essential step in most bacterial infections, and failure to attach leads to eradication of the pathogenic organism. Accordingly, bacteria have evolved sophisticated pili and fimbriae systems for epithelial surface attachment (Mulvey, 2002; Fronzes et al., 2008; Cusumano and Hultgren, 2009), hereby facilitating invasion and colonization of the underlying tissue. Type 1 and p pili are two of such pili systems involved in attachment and invasion of UPEC strains in the host, leading to urinary tract infections. Specifically, Type 1 pili have been implicated in infections of the lower urinary tract, which results in cystitis (infection of the bladder), while p pili are associated with pyelonephritis (infection of the kidney). The p pili and type 1 pili are both consisting of a pilus rod connected to a flexible end tip (fibrillum) that enable interactions with the target.

**Figure 1:**
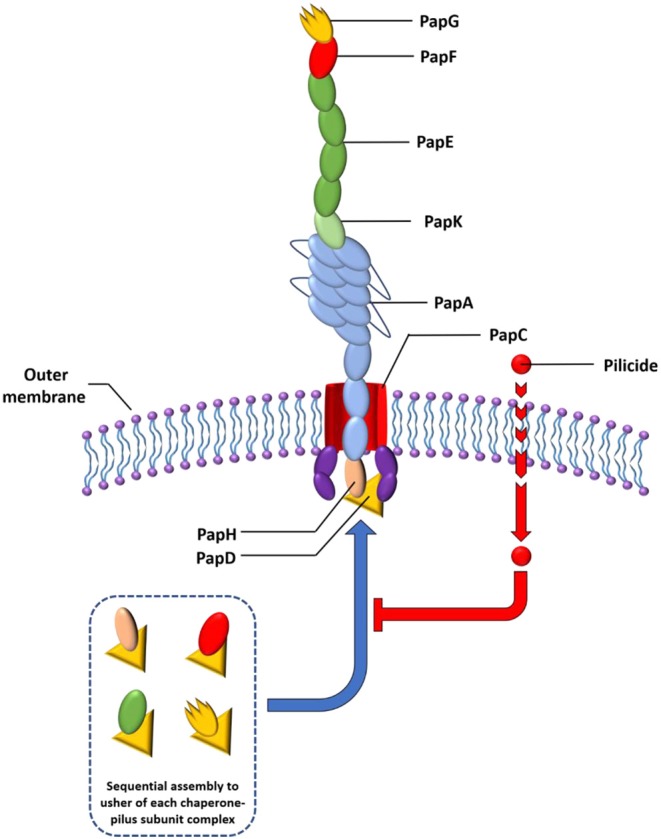
Schematic representation of the P pili. The chaperones attached to the last subunit to be incorporated into each pilus are shown in yellow triangle. P pili are terminated at the outer membrane by the termination subunit, PapH. No such subunit is known in the Fim system. The pilicide bound to the chaperone-pilus subunit complex inhibit interaction with usher for further docking.

Pili are often assembled via a highly conserved mechanism called the chaperone–usher pathway (CUP) utilized by numerous adhesive organelles in Gram negative bacteria, including *E. coli* and *P. aeruginosa* (Figure 1) (Jacob-Dubuisson et al., 1994; Nuccio and Baumler, 2007). The construction of pili by the CUP proceeds from the top to bottom, meaning that the first subunit to be introduced is the adhesin (PapG or FimH), which is followed by introduction of the rest of the tip fibrillum and adaptor subunits (PapF, E, and K or FimG and F), the pilus base (PapA or FimA) and lastly, in the case of the Pap system, the termination and anchor subunit (PapH) (Busch et al., 2015). The CUP pili construction system contains several feasible targets for the development of compounds that block this assembly and thus pili formation.

Among this kind of compounds are mannocides that compete for binding in the mannose binding pocket present in the FimH pilus lectin of type I pili, blocking its binding with their mannose rich receptors in eukaryotic cells. To date, compounds like biphenylmannosides have proved effective *in vitro* to prevent biofilm formation of the UPEC and also to disrupt preformed biofilm (Han et al., 2010). Their oral administration was effective in clearing chronic urinary tract infections in mice and in potentiating the activity of the antibiotic trimethoprim sulfamethoxazole (Cusumano et al., 2011). Bacterial pilus assembly requires the presence of periplasmic chaperones. Thus, development of compounds that bind to chaperones have been of high interest. By inhibiting pilus assembly, such compounds would interfere with bacterial attachment to the host, and therefore they constitute potential novel antibiofilm agents. This type of compounds is referred to as pilicides (Aberg and Almqvist, 2007).

P pili are assembled by the chaperone PapD that binds to and caps interactive surfaces on each pilus subunit thus preventing premature aggregation during their secretion into the periplasmic space. The high degree of structural homology and conserved mechanism of action between the CUP proteins in various pathogens (Rose et al., 2008), make compounds that target this system attractive as potential broad-spectrum anti-virulence compounds.

**Figure 2:**
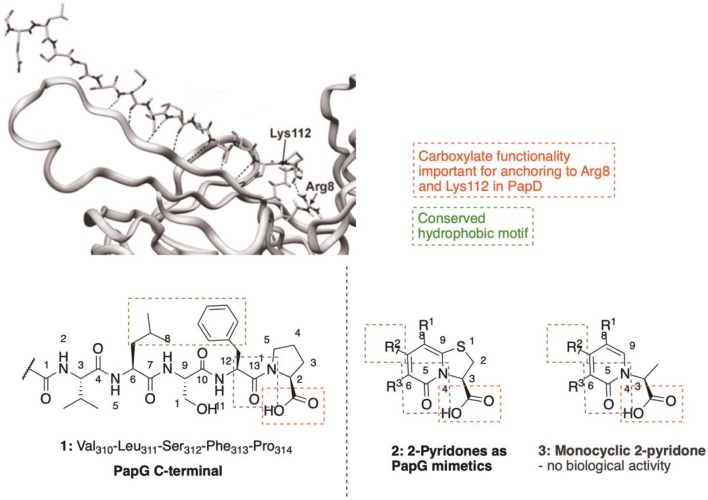
The two final amino acids (Phe_313_-Pro_314_) in the C-terminal of PapG bound to PapD served as template for design of the pilicide scaffold that inhibit pilus assembly.

C-Terminal peptides of both PapG (**1**, Figure 2) and PapH have been shown to bind to the chaperone, leading to effective inhibition of further binding of pilus subunits (Karlsson et al., 1998). This has provided efficient probe molecules useful for studying the intricate processes in the CUPs. However, the use of peptides as drugs has several drawbacks, including poor absorption after oral administration, rapid degradation and/or excretion. Therefore, focus has been given to development of small-molecule peptidomimetics as potential anti-virulence compounds.

The high amount of information available regarding PapG C-terminus interactions with the chaperone (Svensson et al., 2001; Emtenäs et al., 2003a) has been used for a structure-based design of small-molecule structures that mimick the PapG C-terminal. The dihydrothiazolo ring-fused 2-pyridone scaffold (**2**, Figure 2) is the most widely studied pilicide structures, targeting the Arg8/Lys112 cleft region of the chaperone. This scaffold has the terminal carboxylic acid moiety, important for interaction with the Arg8 and Lys112 residues in the chaperone cleft (Figure 2). Furthermore, the scaffold can be further decorated by the installation of substituents at various positions to introduce important hydrophobic motifs (e.g., Leu311 residue) and/or exploit other potentially useful interactions.

The importance of the bicyclic 2-pyridone system was clearly indicated, as the monocyclic 2-pyridone (**3**, Figure 2) showed a considerable lower biological activity as compared to the corresponding bicyclic compound (Pemberton et al., 2008).

**Figure 3:**
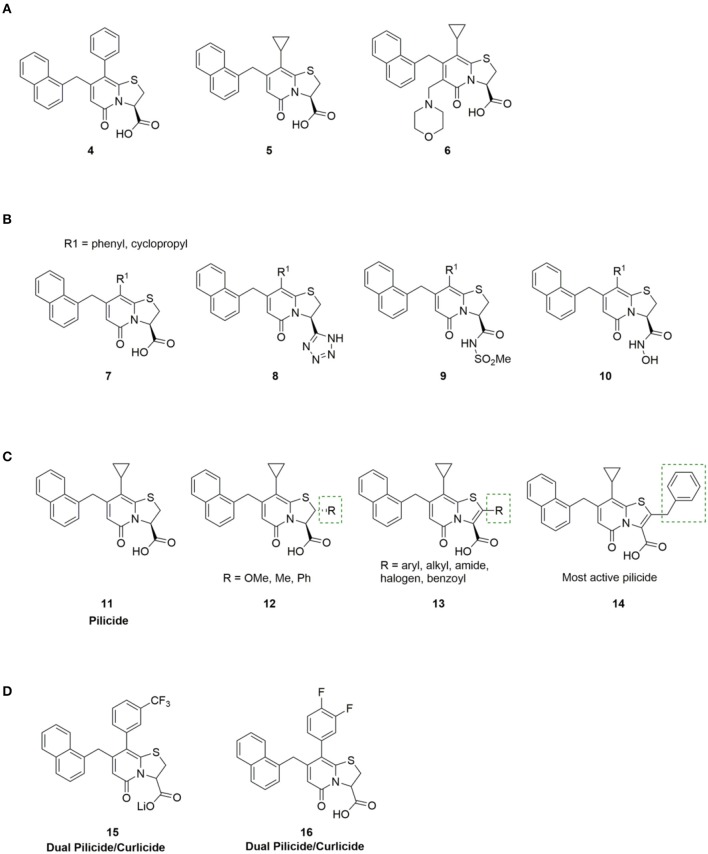
**(A)** First generation pilicides, compound **4** and **5** (Svensson et al., 2001; Lee, 2003) suffered from low solubility. Aminomethylene substitution of pilicide **5** resulted in compound **6** with improved solubility properties (Hedenstrom et al., 2005). **(B)** Pilicides containing carboxylic acid isosteres, including tetrazoles (**8**), acyl sulfonamides (**9**) and hydroxamic acids (**10**) are tolerated (Åberg et al., 2008). **(C)** SAR study to investigate substitution of position C-2 in the pilicide scaffold, including both saturated (**12**) and unsaturated (**13**) analogs (Chorell et al., 2010). **(D)** Small modifications in the phenyl ring allowed identification of ring-fused 2-pyridones that exhibit dual pilicide-curlicide activity (Cegelski et al., 2009).

The first generation bicyclic 2-pyridone pilicides (**4**, **5**, Figure 3) (Svensson et al., 2001; Lee, 2003), suffered from poor water solubility, which limited their utility. However, introducing an aminomethylene substituent in the open C6 position in the 2-pyridone scaffold (**R3**, Figure 2) solved this problem, resulting in 2-pyridone (**6**, Figure 3) binding chaperones in the low millimolar range (Figure 3) (Hedenstrom et al., 2005).

The carboxylic acid group on the pilicide scaffold is vital for the activity of the pilicides, and exchanging the carboxylic acid for other functionalities, including -CO_2_Me, -CH_2_OH, -CH_2_OMe, -CHO, or -CH_3_ substantially reduced the pilicide activity (Aberg et al., 2005a). However, carboxylic acid isosteres comprising tetrazoles (**8**, Figure 3), acyl sulfonamides (**9**, Figure 3) and hydroxamic acids (**10**, Figure 3) are tolerated and can in some cases lead to improved pilicide's potency (Åberg et al., 2008).

The carboxylic acid functionality was originally designed to interact with Arg8 and Lys112 in the chaperone cleft (Figure 2). However, a later NMR-based study elucidating the pilicides' binding site revealed that the cleft of the chaperone is not the only possible binding site (Hedenstrom et al., 2005). Instead, the study suggested that pilicides affect *E. coli* pilus formation either by binding in the chaperone cleft, or by affecting the orientation of the flexible F1–G1 loop of the chaperone, both of which are part of the surface that is involved in interactions with pilus subunits. This latter binding site was later verified by X-ray crystallography of a pilicide-PapD complex (Pinkner et al., 2006).

The effect of varying the C-7 and C-8 (Emtenas et al., 2002; Aberg et al., 2005b; Aberg and Almqvist, 2007; Cegelski et al., 2009; Chorell et al., 2012) position of the 2-pyridone scaffold (Figure 2) have been intensively examined, based on two main objectives. First, to improve the peptide mimicking properties of the identified hits, and secondly to establish structure–activity relationships (SARs) regarding pilicide activity. In general, the nature of the substituent at both the C-7 and C-8 position was found to strongly affect the biological response (Emtenas et al., 2002; Chorell et al., 2012).

In 2011, Chorell et al. conducted a detailed SAR study of the effect of C7 substitution (Chorell et al., 2011). Based on their studies, Almqvist et al. delineated two broad activity trends: (1) Hydrophobic, sterically demanding C-7 substituents are beneficial. (2) Extended ether linkers are allowed and can in some cases improve activity whereas linkers incorporating basic amines, amides and sulphonamides, as well as heteroaryls diminish biofilm inhibition capacity. Overall, the most promising C-7 substituents were found to be 1-naphthylmethyl, naphthoxymethyl, 3-tolylethyl, and 2,3-dimethylphenoxymethyl groups.

Similarly, large and hydrophobic aromatic substituents are important in the R1 position of the 2-pyridinone to get good binding to the chaperone. Compounds containing a phenyl, indole, thiophene or 3,4-methylenedioxyphenyl group at C-8 were effective, while incorporation of pyridine and the smaller methoxy, cyclopropyl, and isopropyl substituents resulted in compounds with reduced potency (Chorell et al., 2012). Furthermore, the R1 substituent has been shown to have an impact on the C2-position in the 2-pyridone (Emtenäs et al., 2003b). For example, 2-pyridone with cyclopropyl at the R1 position was more prone to racemization than 2-pyridone with phenyl at the R1 position.

In 2010 Chorell et al. published a study investigating substitution of the C-2 position in the pilicide scaffold, including both saturated and unsaturated analogs (**12, 13**, Figure 3) (Chorell et al., 2010). As a result of their analysis, Almqvist and co-workers could draw some general conclusions regarding the structural features of the C-2 position in dihydrothiazolo ring-fused 2-pyridone molecules that are necessary for blocking pilus formation. Their results disclosed that the addition of substituents in the C-2 position of the pilicide scaffold significantly enhanced the potency of the pilicides, leading to increased inhibition of pili dependent biofilm formation. However, the effect of the spatial arrangement on activity was different between the analogs. For example, with phenyl substitution in the C-2 position, the unsaturated analog had higher potency than the saturated counterpart, whereas with methyl in the C-2 position, the situation was the opposite; the saturated analog had higher potency than the unsaturated.

On the basis of the results with the unsaturated phenyl analog, a more comprehensive SAR study with unsaturated C-2 aryl and heteroaryl substituents (**13**, Figure 3) was performed. Many of the compounds containing aryl and heteroaryl-substituents efficiently inhibited the formation of pili dependent biofilm. Especially, the benzyl substituted compound **14** (Figure 3) showed promising properties with an estimated EC50 of 7 μM. Interestingly, compounds containing more sterically demanding substituents, including indole and bensodioxane were not tolerated and resulted in no activity.

Almqvist et al. also investigated the importance of the heteroatom in the fused 2-pyridone scaffold, drawing attention to imidazolines and oxazolines. While exchanging the sulfur for a secondary amine resulted in a distinct drop in activity, activities of the oxazolo fused 2-pyridones were comparable to the parent sulfur containing pilicides (Pemberton et al., 2008).

Besides the pili described in the preceding section, *E. coli* and other Enterobacteriaceae produce and display adhesive amyloid fibers termed curli at the bacterial cell surface (Olsen et al., 1989; Kikuchi et al., 2005). Curli are critical for biofilm development in *E. coli*, making this extracellular macromolecule a highly relevant anti-biofilm target. Assembly of curli depends upon at least six proteins known as CsgA, CsgB, CsgD, CsgE, CsgF, and CsgG. CsgA and CsgB are the major and minor curli subunits, respectively, while CsgE, CsgF, and CsgG are accessory proteins that direct the extracellular localization and facilitate assembly of curli subunits into fibers.

Curlicides designate compounds that inhibit uropathogenic *E. coli* curli biogenesis and prevent the polymerization of CsgA; being important for anchoring the curli amyloid fiber to the bacterial outer membrane (Hammar et al., 1996). Thiazolo ring-fused 2-pyridones are peptidomimetics that target important protein-protein interactions in macromolecular assembly (Svensson et al., 2001; Emtenäs et al., 2003a), which, as discussed above, provides an excellent scaffold for development of pilicides (for example **11**, Figure 3). However, their ability to disrupt protein-protein interactions has also been investigated to identify scaffolds that show inhibitory effects on curli biogenesis (Aberg et al., 2005b). While the cyclopropyl-substituted pyridone **11** (Figure 3) with strong pilicide activity, exerted no anti-curli activity (Cegelski et al., 2009), exchange of the cyclopropyl group of **11** with larger aryl substituents generated compound **15** and **16** (Figure 3) that gained curlicide activity and blocked curli-dependent biofilms. Interestingly, **15** and **16** both retained their ability to prevent pili formation, thus exhibiting dual pilicide-curlicide activity (Cegelski et al., 2009). Compounds that can act as both pilicides and curlicides could have increased therapeutic value as they could inhibit the formation of several adhesion fibers that are important in biofilm formation (Cegelski et al., 2009).

### Modulators of c-di-GMP Signaling in *Pseudomonas aeruginosa* and *Escherichia coli*

**Figure 4:**
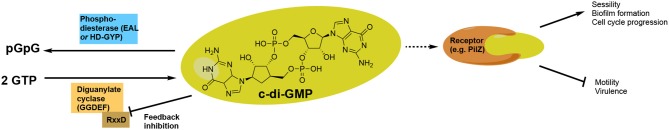
Graphical illustration of c-di-GMP signaling.

Cyclic di-GMP has emerged as an almost universal positive regulator of biofilm formation in Gram negative bacteria (Figure 4) (Jenal et al., 2017). There are two basic concepts for external intervention that reduces the c-di-GMP level in bacteria: decreasing c-di-GMP formation by inhibition of DGCs or increasing c-di-GMP degradation by activation of PDEs.

DGCs contain GGDEF catalytic domains and function as homodimers. The GGDEF domains are placed at the interface of the dimer and are important for the binding of two molecules of GTP as well as their conversion into c-di-GMP with Mg^2+^ functioning as cofactor (Valentini and Filloux, 2016). Some DGC proteins, such as PelD from *C. crescentus*, WspR from *P. aeruginosa*, and YdaM from *E. coli* as well as DgcK and DgcL from *Vibro cholera*, possess an inhibitory site (I-site), placed only five amino acids away from the active site (Valentini and Filloux, 2016). A characteristic motif of the I-site is an RxxD sequence (x is any amino acid), where the c-di-GMP product can bind to, thereby allostericcally inhibiting its own synthesis (Chan et al., 2004; Kalia et al., 2013).

So far, two main types of c-di-GMP PDEs have been characterized, containing either the EAL or HD-GYP domain (Schirmer and Jenal, 2009; Romling et al., 2013). The primary role of EAL domain PDEs is to linearize c-di-GMP into 5′-phosphoguanylyl-guanosine (5′-pGpG) and only slowly hydrolyze 5′-pGpG to GMP. EAL-domain-containing proteins require Mg^2+^ or Mn^2+^ ions for catalysis, while inhibited by Ca^2+^ (Schmidt et al., 2005). The HD-GYP domain-containing PDEs are the second group of c-di-GMP specific PDEs, functioning by hydrolyzing c-di-GMP directly into two GMP molecules. Like the EAL domain proteins, the HD-GYP domain proteins have a binuclear Fe^2+^ or Mn^2+^ center (Romling et al., 2013).

Due to the highly conserved nature of c-di-GMP signaling systems in bacteria, and the strong evidence for their role in regulating biofilm formation, targeting c-di-GMP signaling systems is a promising approach for development of broad-spectrum small molecules for treatment of biofilm-associated infections.

Both PDEs and DGCs interact extensively with c-di-GMP, making it challenging to design c-di-GMP analogs that selectively target PDEs and not DGCs, and vice versa. In addition, most bacteria synthesize multiple DGCs and PDEs, each of which affect distinct phenotypes (Opoku-Temeng and Sintim, 2017). For example, Lory et al. observed that *P. aeruginosa* overexpressing the DGC PA2870, and other GGDEF domain proteins such as SiaD and PA0575, showed no change in ability to form biofilm, while *P. aeruginosa* overexpressing the DGCs WspR, RoeA, and PA3702 showed increased biofilm formation (Kulasakara et al., 2006). Just like DGCs, not all PDEs affect the global concentrations of c-di-GMP but regulate other processes, e.g., the synthesis of virulence factors (Opoku-Temeng and Sintim, 2017) Therefore, targeting inhibition of some of these virulence-associated PDEs (as long as they do not regulate biofilm dispersal) with small molecules could be pursued for blockage of virulence. However, the complexity of the c-di-GMP signaling pathway is challenging when developing small molecule modulators. Nonetheless, some progress has been made and several inhibitors of c-di-GMP metabolizing enzymes that affect biofilm formation and motility have been described. Here we discuss various reported inhibitors of c-di-GMP signaling.

### Small Molecule Inhibitors

**Figure 5:**
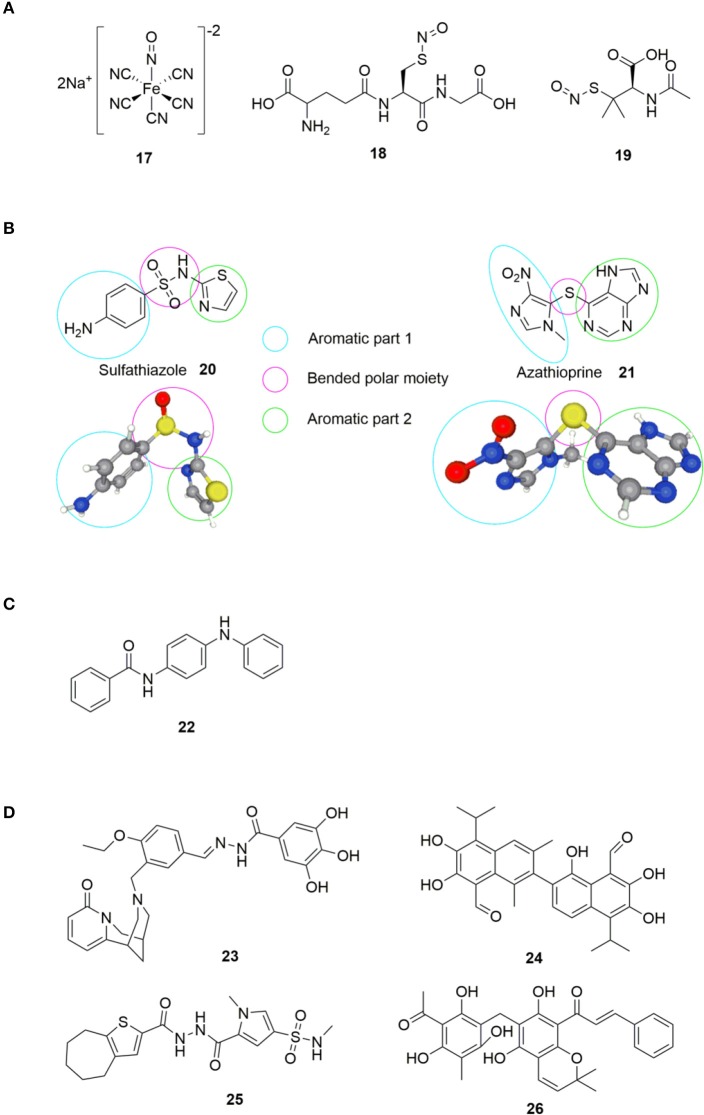
**(A)** NO donors exerting biofilm-inhibitory effect (Barraud et al., 2006). **(B)** Sulfathiazole **20** and azathioprine **21**; small molecules found to interfere with c-di-GMP biosynthesis by the group of Antoniani et al. (2010, 2013). **(C)** Compound identified as DGC inhibitor (DI) by Sambanthamoorthy et al. (2014). **(D)** Inhibitors of DGC discovered by Sambanthamoorthy et al. (2014).

The history of small molecule DGC-inhibitors started in 2006 with work by Webb and colleagues, who discovered that the molecule nitric oxide (NO) can induce dispersal of *P. aeruginosa* biofilms (Barraud et al., 2006). The authors suggested a combined treatment approach with a NO donor and an antimicrobial agent to eradicate biofilm infections. They examined various NO donors, specifically sodium nitroprusside (SNP) **17**, S-nitroso-L-glutathione (GSNO) **18** and S-nitroso-N-acetylpenicillamine (SNAP) **19** (Figure 5) in combination with the antimicrobial agents tobramycin, hydrogen peroxide, and sodium dodecyl sulfate. Of the investigated NO donors, SNP was shown to be the most potent inhibitor of biofilm formation in *P. aeruginosa* (Barraud et al., 2006).

Although NO-donors were shown to exert biofilm inhibitory effects, it was not until 2009 that the biochemical understanding of this effect was revealed (Barraud et al., 2009). Kjelleberg et al. showed that low nontoxic levels of NO stimulated PDE activity in *P. aeruginosa*, causing an overall decrease in intracellular c-di-GMP, leading to biofilm-dispersal. Besides the reduction of the intracellular c-di-GMP level, NO-donors cause a downregulation of the synthesis of pyoverdine (Kang et al., 2017), which is a siderophore responsible for recruitment of essential iron for biofilm formation (Kang and Kirienko, 2017).

In 2010, Antoniani et al. developed an assay for high throughput screening of DGC-inhibitors. They screened a library of 1120 compounds, which allowed them to identify the antimicrobial sulfathiazole **20** (Figure 5), as an efficient inhibitor of the *E. coli* DGC-protein AdrA (Antoniani et al., 2010). Sulfathiazole consists of a free aniline, an aromatic sulfonamide and a thiazole ring, all polar and rather electron rich components. This results in a molecular structure with two planar sides that is bended around the sulfonamide moiety (Figure 5). Subsequently, Landini et al. provided evidence that azathioprine **21** (Figure 5), an anti-inflammatory drug used in treatment of several autoimmune conditions, can impede c-di-GMP biosynthesis in *E. coli* (Antoniani et al., 2013).

Comparing azathioprine **21** to sulfathiazole **20** (Figure 5), they share structural similarities with to (hetero) aromatic parts bended around a polar moiety (sulfide and sulfonamide, respectively). Sulfathiazole **20** and azathioprine **21** (Figure 5) do not interfere with the c-di-GMP biosynthesis through inhibition of DGC activity. Instead, sulfathiazole **20** and azathioprine **21** was shown to affect nucleotide metabolism, leading to an alteration of nucleotide pools, hereby affecting c-di-GMP substrate availability.

In 2012, Sambanthamoorthy et al. published the screening of approx. 66.000 compounds for inhibition of DGC activity in *Vibrio cholerae* (Sambanthamoorthy et al., 2012). After several rounds of screening and optimization, 8 compounds were identified with good antagonistic effect toward multiple DGC enzymes, with N-(4-anilinophenyl)benzamide **22** (Figure 5) showing significant reduction of biofilm formation also in *P. aeruginosa*.

In 2014, Palys et al. reported an *in silico* pharmacophore-based screening of ~15.000 small molecules for their ability to inhibit DGC and control biofilm development (Sambanthamoorthy et al., 2014). Four compounds, LP 3134 **23**, LP 3145 **24**, LP 4010 **25**, and LP 1062 **26** (Figure 5), were identified that significantly reduced WspR activity in *P. aeruginosa*. Further studies revealed that the compounds significantly prevented biofilm formation by *P. aeruginosa* and *A. baumannii* in a continuous-flow system (Sambanthamoorthy et al., 2014). All four molecules were found to disperse biofilm in *P. aeruginosa* and inhibited biofilm development on urinary catheters, whereas only one of the molecules (**23**) dispersed *A. baumannii* biofilms.

**Figure 6:**
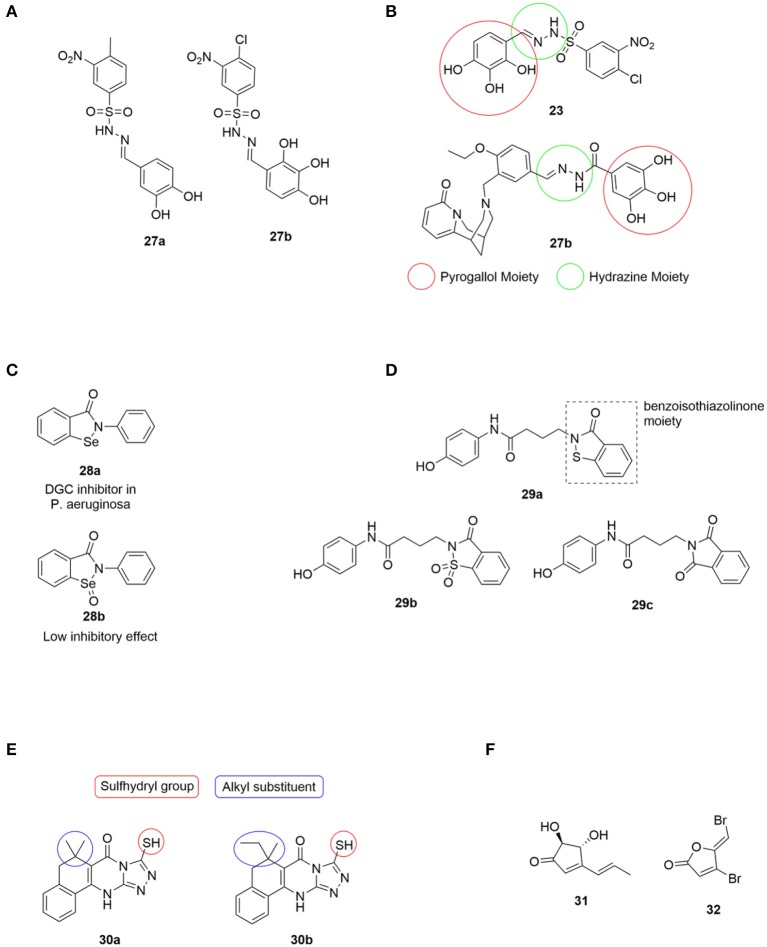
**(A)** DGC inhibitors investigated by Rinaldo et al., all significantly reduced PleD activity. Amb379455 **27b** being the most promising (Fernicola et al., 2016). **(B)** Structual comparison of LP3134 **23** and Amb379455 **27b. (C)** The small molecule ebselen (Eb) **28a** identified as an DGC inhibitor by Lee and coworkers, and its oxidized form ebselen oxide (EbO) **28b** (Lieberman et al., 2014). **(D) 29a** discovered by the Sintim group, and further investigated the importance of the benzoisothiazolinone moiety by testing **29b** and **29c** (Zheng et al., 2016). **(E)** H19 **30a** and 925 **30b**; two Alg44 (pilZ domain) inhibitors identified by Wang et al., and structural features further investigated by (Zhou et al., 2017). **(F)** The small molecules terrein **31** and **32** used by Kim et al. in studying the correlation between regulation of c-di-GMP levels and QS.

A virtual screening approach was also undertaken by Rinaldo and coworkers, who reported the *in silico* screening of ~2.3·10^7^ compounds from the ZINC database, with the aim to identify potent DGC inhibitors targeting the active site (Fernicola et al., 2016). The active site of the DGC PleD from *C. crescentus* was used as structural template. Seven of the tested compounds showed significant reduction in PleD DGC activity, with Amb2250085 **27a** and Amb379455 **27b** (Figure 6) both containing the sulfonohydrazide, the nitro-group and the catechol moiety, being the most efficient PleD inhibitors. Further studies revealed that **27a** was very sensitive to metals, such as Mg^2+^, which completely abolished the inhibitory activity of both PleD, WspR and YfiN. However, **27b** proved not to be interfered by the presence of the divalent metal ion. **27b** showed strong inhibition of DGCs, including WspR and YfiN from *P. aeruginosa*, through binding to the active site of the DGCs.

It is relevant to notice the structural similarity when comparing Amb379455 **27b** and the previously mentioned LP 3134 **23**, as illustrated in Figure 6. Both DGC inhibitors share the *N*-benzylidenebenzohydrazide moiety that is predicted to interact with an amino group in the active site (the Asn335 residue). Furthermore, they both contain the pyrogallol unit, a moiety with high polarity and strong coordination properties. Polyphenols characteristically possess a significant binding affinity for proteins (Papadopoulou and Frazier, 2004). Furthermore, pyrogallol can undergo reversible oxidation, to generate reactive oxygen species (ROS), resulting in DNA damage (Beaber et al., 2004; Kohanski et al., 2007). Worth mentioning, evidence suggests that polyphenolic compounds can interfere with bacterial QS by blocking AHL-mediated signaling between bacteria (Huber et al., 2003).

In 2014, Lieberman et al. identified the small molecule, ebselen (Eb) **28a** (Figure 6), as an inhibitor of allosteric binding of c-di-GMP to receptors containing an RxxD domain, including WspR and PelD of *P. aeruginosa* (Lieberman et al., 2014). It was found that Eb **28a** covalently modified DGCs by forming a bond between the selenium in Eb **28a** and a thiol in a cysteine residue in the allosteric inhibitory site. It was further demonstrated that the allosteric inhibition of DGCs also inhibited the activity of the DGCs. The oxidation state of selenium was important for activity, as the corresponding ebselen oxide (EbO) **28b** (Figure 6) showed reduced inhibition.

In 2016, Sintim et al., screened 250.000 compounds in order to find a small molecule PDE inhibitor that bind to the EAL domain of YahA in *E. coli* (Zheng et al., 2016). A handful of such PDE binders were identified and tested for their inhibitory effect of RocR (from *P. aeruginosa*) with only the benzoisothiazolinone derivative **29a** (Figure 6) successfully inhibiting the hydrolysis of c-di-GMP. **29a** was tested against a series of PDEs from different bacteria and shown to be highly selective toward inhibition of RocR. The importance of the benzoisothiazolinone moiety was clearly indicated as **29b** and **29c** (Figure 6) showed a dramatic decrease in inhibitory effect. RocR is an example of a virulence-associated PDE that does not affect the global c-di-GMP level in *P. aeruginosa* and therefore, inhibition of RocR may hold potential for inhibiting virulence. **29a** was the first example of cell-permeable PDE inhibitors that selectively inhibits one type of PDE without affecting other PDEs. The tested PDEs were YahA (*E. coli*), DipA (*P. aeruginosa*), PvrR (*P. aeruginosa*), PA4108 (*P. aeruginosa*), and RocR (*P. aeruginosa*). Furthermore, they showed no effect on the DGCs WspR and D70E from *P. aeruginosa*.

In 2017, Wang et al. identified two compounds, H19 **30a** and 925 **30b** (Figure 6) that interrupted c-di-GMP binding to Alg44 (PilZ domain) in *P. aeruginosa*, thereby inhibiting its ability to produce alginate, an exopolysaccharide polymer which is part of the biofilm matrix (Zhou et al., 2017). The sulfhydryl group, forming a covalent disulfide bond with Cys-98 in Alg44 (Figure 6), was found to be of high importance for activity. Furthermore, a broad range of alkyl substituents was tested without any improvement of inhibitory activity (Zhou et al., 2017).

In 2018, Kim et al. published a study that suggested a connection between c-di-GMP signaling and QS in *P. aeruginosa* (Kim et al., 2018). Terrein **31** (Figure 6) was found to inhibit biofilm formation by blocking QS receptors, similar to the effect observed for furanone C-30 **32** (Costas et al., 2015). However, terrein **31** was found to decrease c-di-GMP levels; while furanone C-30 **32** increased it. The study of Kim et al. suggested that the QS system controls the c-di-GMP levels by regulating the activity of DGCs and PDEs. DGC activity seemed to be regulated by Las-QS via LasR and PDE by Rhl-QS (Costas et al., 2015).

### Substrate and/or Product Analogs

Another approach for development of enzyme inhibitors is based on compounds that mimic the electronic and/or geometric characteristics of the substrate(s) or product(s), inducing enzyme inhibition due to competitive binding or a negative feedback loop, respectively.

**Figure 7:**
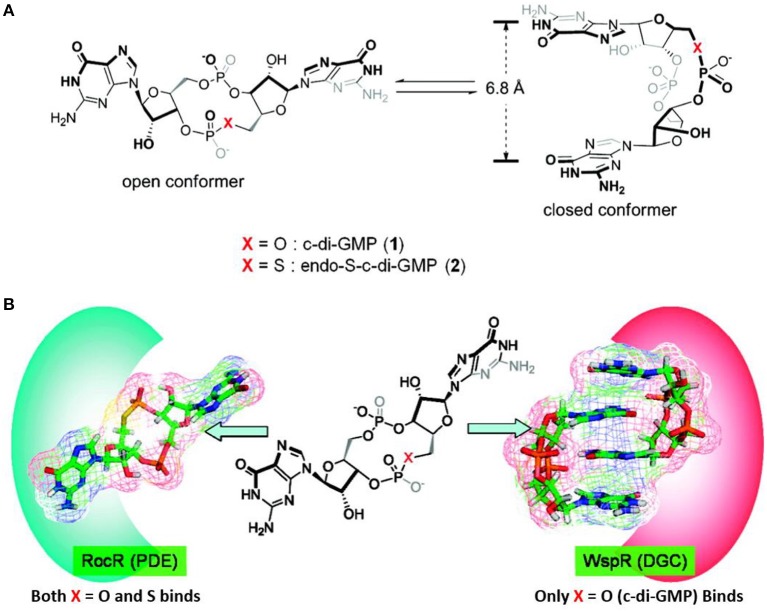
Illustration of the open and closed conformation of X-modified c-di-GMP by Wang et al. (2011). **(A)** Without enzymes **(B)** with enzymes illustrated.

In 2011 Sintim et al. performed the first of many studies on analogs of c-di-GMP (Wang et al., 2011). They studied the two conformations (open and closed) of c-di-GMP (Figure 7) and investigated how changing the bridging heteroatom X could shift the equilibrium between the conformations. C-di-GMP binds to PDEs in an open conformation, while it binds to DGCs in a closed conformation as a dimer (Figure 7) (Wang et al., 2011). Therefore, developing c-di-GMP analogs that prefers an “open” vs. “closed” conformer could allow for a control of the binding affinity toward PDEs vs. DGCs.

**Figure 8:**
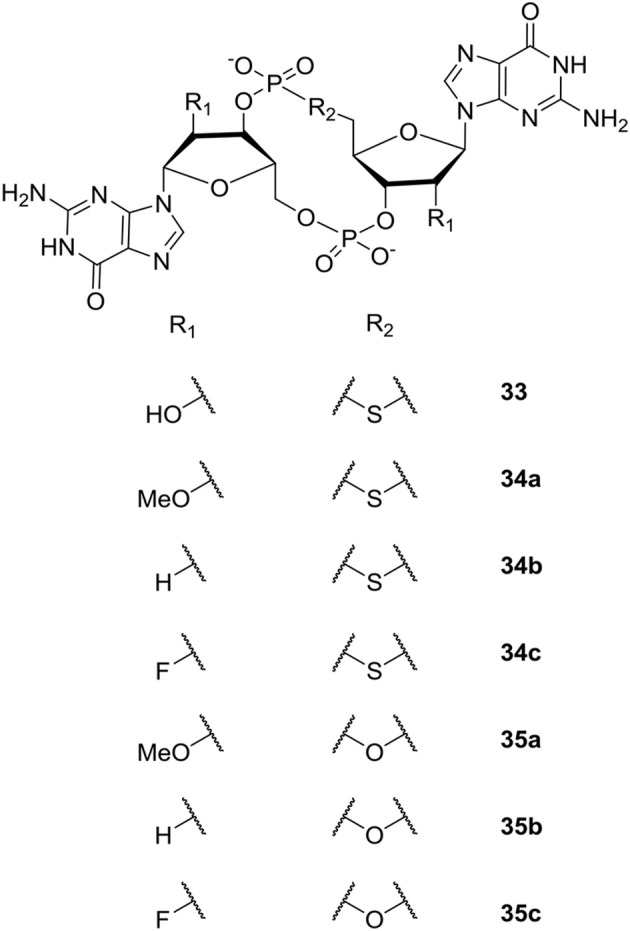
C-di-GMP analogs studied by the group of Sintim (Wang et al., 2011; Zhou et al., 2012, 2013).

The study by Sintim et al. revealed that the sulfur-substituted analog endo-S-c-di-GMP **33** (Figure 8) that predominantly stay in an open form, selectively inhibited PDEs without affecting the potent *P. aeruginosa* DGC WspR. Although not directly relevant for inhibition of biofilm, the work nicely demonstrated that selective binding to specific classes of c-di-GMP binding proteins can be accomplished by altering conformer populations of the analog (so-called conformational steering). This could be utilized as design principle when developing selective PDE-activators or DGC-inhibitors that interfere with biofilm formation and/or persistence. Furthermore, as discussed previously, selective inhibition of some of the virulence-associated PDEs that do not regulate biofilm dispersal could be relevant for treatment of biofilm-associated infections.

Subsequently, the Sintim group expanded their studies of c-di-GMP analogs to include modification of the 2′-position of the ribose moiety (Zhou et al., 2012, 2013). Both analogs bridged with sulfur (Zhou et al., 2012) and oxygen (Zhou et al., 2013) were studied (see Figure 8). Biophysical characterization and comparison of relative energies showed that endo-S-c-di-GMP **33** and the flour-substituted endo-S-c-di-GMP analog (2′-F-endo-S-c-di-GMP **34c**, Figure 8) preferred the closed form, whereas the methoxy-substituted 2′-OMe-endo-S-c-di-GMP **34a** (Figure 8) preferred the open form. In comparison, the 2′-H-endo-S-c-di-GMP **34b** (Figure 8) showed similar energies for both conformations. Sintim et al. then investigated the binding of the c-di-GMP analogs to a Vc2 RNA riboswitch. So far, two classes of riboswitches that binds c-di-GMP have been identified; the c-di-GMP-I riboswitch (class I) and c-di-GMP-II riboswitch (class II). The Vc2 RNA is a class I riboswitch, and crystal structure studies have shown that class I riboswitches bind c-di-GMP in the closed conformation. Of the new analogs, the flour-substituted 2′-F-endo-S-c-di-GMP **34c** had the highest relative binding to the class I riboswitch. Although more enzyme-selective, the binding affinity was similar to the binding affinity of native c-di-GMP and endo-S-c-di-GMP **33** (Figure 8). The investigations showed that the 2′-position is essential for binding to the class I riboswitch, Vc2 RNA, which can be used to design analogs that only target specific c-di-GMP proteins.

Studying the ribose-modified analogs bridged with oxygen (Zhou et al., 2013) (Figure 8), Sintim et al. found that the 2′-F-substituted ribose analogs (c-di-2′F-GMP **35c**) bind to the I-site of DGC four times better than c-di-GMP, while c-di-GMP bound 10 times better to PDEs than c-di-2′F-GMP **35c**. Although, the c-di-2′F-GMP **35c** was seen to still inhibit PDEs, their results showed the potential of designing selective DGC-inhibitors through investigation of I-site binders. The bulky 2′-OMe-substituted **35a** analog was found to be a poor DGC-inhibitor, which they explained with lack of space in the binding pocket of the enzymes tested. The polarity reduced 2′-H-substituted analog **35b** was shown to have similar binding-affinity as c-di-GMP.

**Figure 9:**
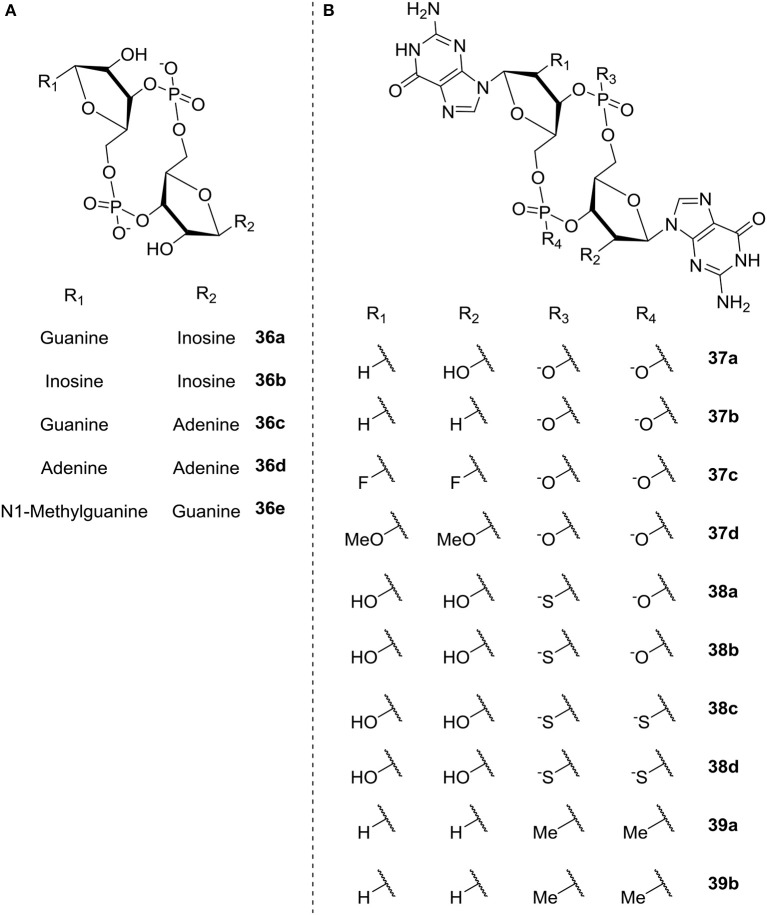
C-di-GMP analogs studied by Shanahan et al. (2013) **(A)** base modification (**36a-e**) **(B)** ribose modification (**37a-d**), phosphate modification (**38a-d**), both ribose- and phosphate-modification (**39a-b**).

Shanahan et al. (2013) also studied c-di-GMP analogs; here with the aim of identifying analogs that were resistant to hydrolysis by PDE, in order to apply them for riboswitch control. The different analogs studied are illustrated in Figure 9 and divided into two groups according to the modification; base modification (**A**, **36a-e**) and ribose- and phosphate modification (**B**, ribose **37a-d**, phosphate **38a-d**, both **39a-b**), respectively. The authors evaluated the rate of hydrolysis by PDE as compared to c-di-GMP. In the three categories base, ribose and phosphate, the latter gave the best overall results. They found that substitution of the phosphates with phosphorothioatea (e.g., c-(R_p_R_p_)-di-G_ps_ **38c** and c-(R_p_S_p_)-di-G_ps_, **38d**, Figure 9) yielded analogs with good binding affinity toward both classes of riboswitches (Shanahan et al., 2011). Additionally the thio-substituted analogs showed good binding affinity toward the EAL domain without being degraded; indicating their potential as PDE-inhibitors.

**Figure 10:**
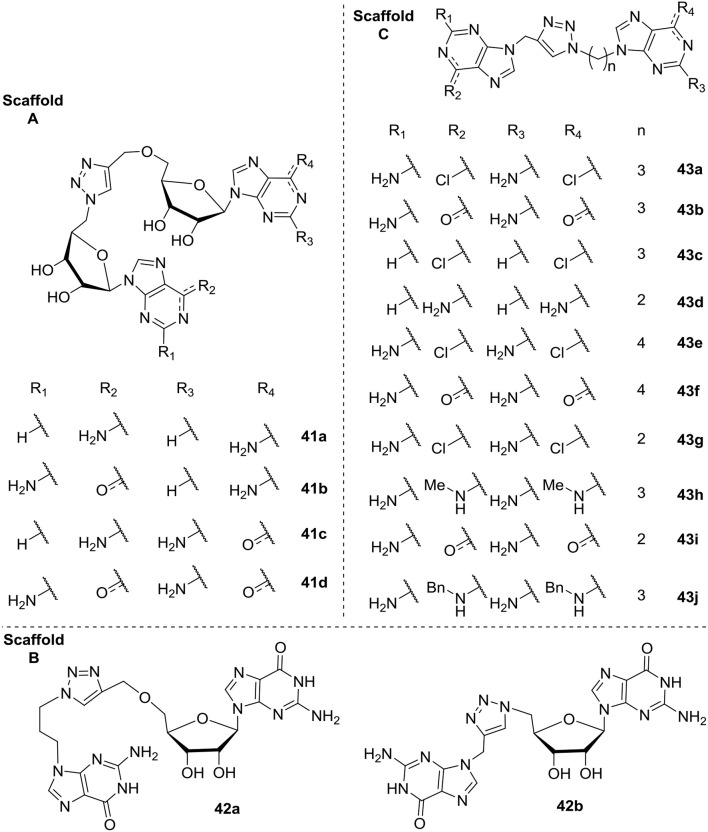
C-di-GMP analogs, with a 1,2,3-trizole moiety incorporated, investigated for their allosteric inhibitory effect on DGC by Cutruzzolá et al. The analogs are grouped into three Scaffold types (**A–C**; Fernicola et al., 2015).

In 2015 Cutruzzolá et al. investigated c-di-GMP analogs to identify allosteric inhibitors of DGCs that does not affect PDEs (Fernicola et al., 2015). They synthesized and screened a series of 16 analogs where the phosphate/ribose moiety was replaced with a non-hydrolyzable 1,2,3-triazole. Different analogs were investigated with variation of the guanine base moiety (Scaffold A, Figure 10), as well as analogs where one (Scaffold B, Figure 10) or both (Scaffold C, Figure 10) of the two ribose-moieties was omitted. Of the tested analogs, the simple analog DCI061 **43b** (Scaffold C, Figure 10) showed good inhibition of the *P. aeruginosa* PDE; RocR. When further investigating the DCI061(**43b**)-scaffold, Fernicola et al. were able to draw some general conclusions: (1) Introducing bulky substituents in the guanine base, resulted in a dramatic loss of inhibitory activity toward RocR. (2) The electrophilic character of the substituent in the C-6 position proved to be crucial for binding to the inhibitory site of DGCs and anti-binding to the EAL active site (PDEs). (3) The 2-amino group in guanine is important for binding of DGC, while not crucial for binding to EAL. (4) The distance between the two purines was crucial, with a 3-carbon spacer resulting in optimal interaction, while both a 2- and 4-carbon spacer resulted in complete loss of inhibitory activity.

**Figure 11:**
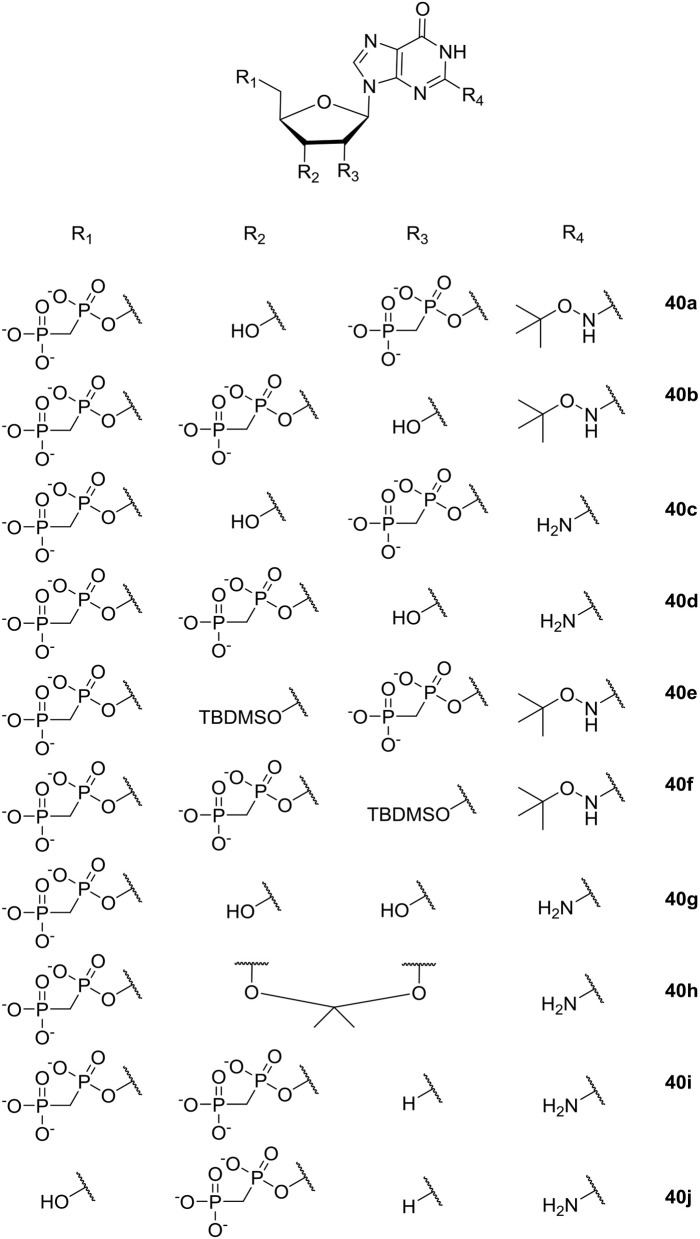
ppGpp analogs studied by Wexselblatt et al. (2010).

Guanosin-tetraphosphate (ppGpp), i.e., phosphorylated analogs of GTP and GDP, is produced by the Rel proteins and accumulates in a variety of bacterial species as a stress response to amino acid starvation. Under these conditions amino acids are maintained and RNA synthesis is inhibited; causing, among other effects, low ability to sustain biofilm formation (Wexselblatt et al., 2010). Therefore, inhibition of the synthetic activity of Rel proteins may prevent bacteria from sensing conditions where amino-acids are absent in their environment, which, in turn, may ultimately lead to bacterial self-starvation and death. In 2010 Wexselblatt et al. studied ppGpp analogs (see Figure 11) (Wexselblatt et al., 2010), and tested them as competitive inhibitors of Rel proteins. Vidavski et al. observed that 2′-deoxyguanosine-3′-5′-di(methylene bisphosphonate) (**40i**, Figure 11) is a competitive binder for GTP in the Rel protein; causing inhibition of the protein and lowering of ppGpp production.

### Inhibitors of QS in *Pseudomonas aeruginosa* and *Acinetobacter baumannii*

This section presents an overview of small molecules identified as QS inhibitors (QSIs) targeting *P. aeruginosa* and *A. baumannii*. QS inhibition is a way to attenuate pathogenicity without obstructing processes that are essential for bacterial growth. In this section we will focus on compounds targeting the AI-1 system used by both *P. aeruginosa* and *A. baumannii* and not cover molecules targeting the PQS system only used by *P. aeruginosa*. Several comprehensive reviews about QS inhibitors have been published, some of which focus on the biological activity of the compounds (Jakobsen et al., 2013, 2017a; Kalia, 2013) and others on the structures providing knowledge as to the molecular characteristics important for inhibition (Geske et al., 2008b; Galloway et al., 2011). *P. aeruginosa* is by far the most studied organism in relation to inhibition of AHL-mediated QS, whereas the number of studies investigating QS inhibition of *A. baumannii* is more limited.

The quest for small molecules capable of lowering the efficacies of QS systems has gained considerable attention for more than two decades, and it has resulted in the identification of a large variety of QS inhibitors. It has been shown that a QS-deficient *P. aeruginosa* biofilm established either by the use of QS deficient mutants or treatment with QS inhibitors shows a decrease in survival in a mouse lung infection model compared to a *P. aeruginosa* infection with a functional QS system (Wu et al., 2004; Bjarnsholt et al., 2005; Jakobsen et al., 2012b). Three factors that are most likely involved in this change in *P. aeruginosa* are rhamnolipids, extracellular DNA and pyocyanin. They are all partially or completely QS regulated and are shown to provide protection from host immunity as well as antibiotic therapy (Davey et al., 2003; Allesen-Holm et al., 2006; Jensen et al., 2007; Chiang et al., 2013; Das et al., 2015).

### Modulators of AHL-Based QS Systems

**Figure 12:**
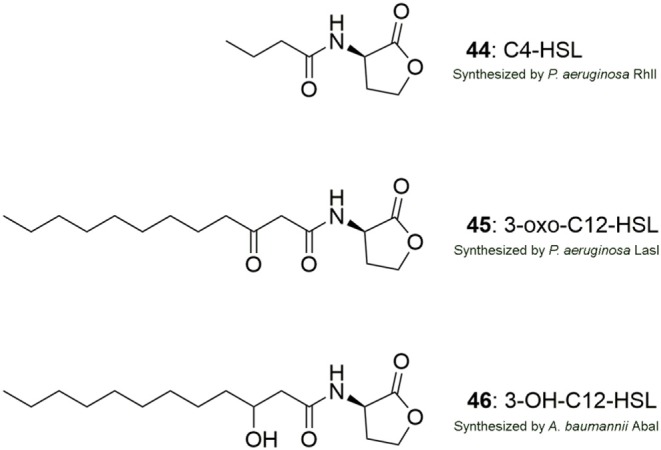
Structures of the AHL signal molecules produced by *P. aeruginosa* (**44**, **45**) and *A. baumannii* (**46**).

A general perspective is that there are three targets to chemically affect AHL mediated QS systems by small molecules: (I) the signal production (LuxI-type synthase), (II) the signal molecule (AHL-ligand) and (III) the signal receptor (LuxR-type receptor) (Rasmussen and Givskov, 2006). However, this is a perspective with modifications and differences between the AHL QS systems in bacterial organisms exist. For instance, the AHL QS system in *P. aeruginosa* consists of two LuxIR-type systems, LasIR and RhlIR, which are interlinked, contrary to the QS system in *A. baumannii* which is based on only a single LuxIR system, AbaIR. The structure of the signal molecules employed by these QS systems is shown in Figure 12. In addition, the *P. aeruginosa* QS system is comprised of additional systems such as IQS and PQS connected to the Las and the Rhl system making the inhibition of the system more complex.

**Figure 13:**
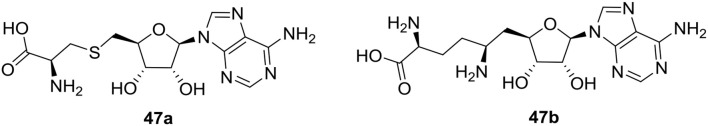
Structures of S-adenosyl-homocysteine (**47a**) and sinefungin (**47b**).

Inhibition of the AHL synthase can be an effective strategy to disconnect cell to cell signaling, however at present there are very few studies with a thorough investigation of small molecules targeting the signal production. Studies have investigated the potential of using analogs of S-adenosylmethionine (SAM), which is the amino donor for the formation of the homoserine lactone ring moiety. Three analogs, S-adenosyl-homocysteine **47a**, sinefungin **47b** (Figure 13) and butyryl SAM have been shown to inhibit *P. aeruginosa* RhlI synthase and block AHL production *in vitro* (Parsek et al., 1999).

Targeting QS signaling directly by enzymatic degradation of the signal molecule can be achieved by an AHL-lactonase, AHL-acylase or a paraoxonase that break the AHL molecule or by oxidoreductases that reduce the carbonyl to a hydroxyl group (Chen et al., 2013). Several AHL-lactonases have been discovered to date including the enzyme MomL that was identified from *Muricauda olearia* Th120. MomL was shown to efficiently degrade a number of different AHLs (Tang et al., 2015). Recently, MomL was shown to reduce biofilm formation of *P. aeruginosa* and *A. baumannii* as well as to increase biofilm susceptibility to different antibiotics. However, no activity was detectable in a wound biofilm model or in a *C. elegans* model questioning the *in vivo* anti-QS effect of MomL (Zhang et al., 2017). AiiA lactonase isolated from the soil bacterium *Bacillus* sp. 240B1 was the first lactonase to be described (Dong et al., 2000). Later the AiiM lactonase was shown to be an effective inhibitor of *P. aeruginosa* QS and to reduce a *P. aeruginosa* infection in a mouse model of acute pneumonia (Migiyama et al., 2013). Paraoxonases (PON) has been identified in several different mammals. They have strong degradation activity toward the acylated chain of AHLs especially if the chain is long. The *P. aeruginosa* signal molecule 3-oxo-C12-HSL is effectively hydrolyzed by PON resulting in attenuation of the QS system (Chun et al., 2004; Yang et al., 2005; Teiber et al., 2008).

Most studies investigating QS inhibition by small molecules have been focused upon identification of compounds that interact and paralyze the LuxR-homolog receptor. By using AHL analogs that match the LuxR-homolog, a signal-receptor complex is generated that is competitive to the native active complex leading to disruption of signaling. Several studies have successfully identified a range of both QS activators and inhibitors by introducing changes to the lactone moiety or the acyl chain of the native signal molecules. The generated X-ray crystal structure of some LuxR-type receptors in a complex with their natural AHL ligands has given the opportunity to use this information in the design of synthetic AHL ligands. The crystal structure of the ligand-binding domain of LasR has been solved (Bottomley et al., 2007), whereas no structure of AbaR has been reported to date. In addition, to the search for AHL analogs there has been a significant effort to identify small molecules that are distinct from the native AHL molecules. The commonly used methods have been screening of random chemical libraries consisting of either natural products isolates or synthetic made compounds together with bioassay-guided fractionation of natural products.

### Studies on AHL-Derived QS Modulators

**Figure 14:**
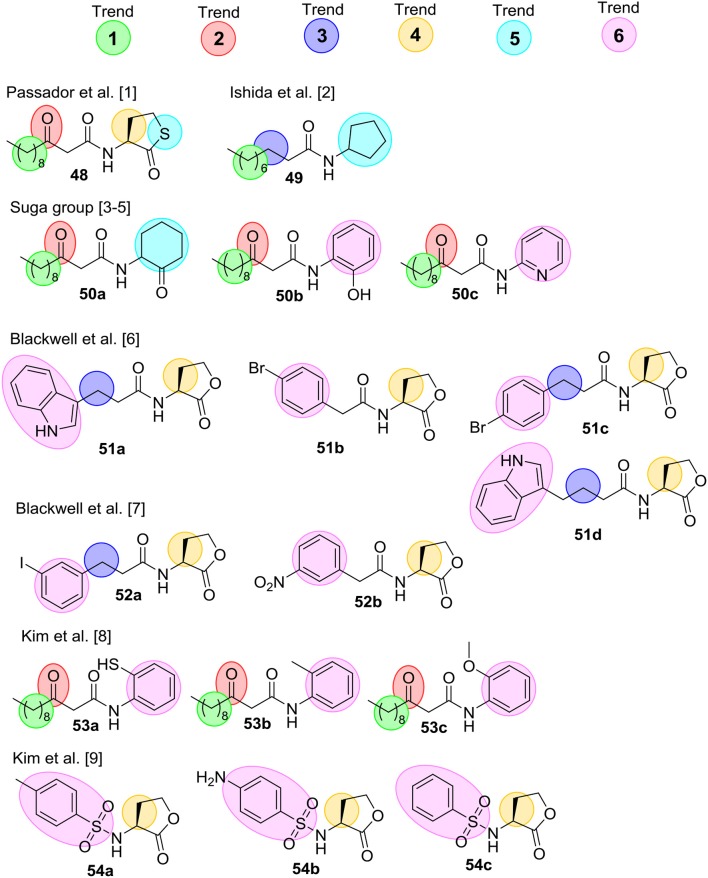
Collection of the most potential QS inhibitors mentioned in Geske et al. (2008b) and Galloway et al. (2011). [1] (Passador et al., 1996) [2] (Ishida et al., 2007) [3] (Smith et al., 2003a) [4] (Smith et al., 2003b) [5] (Jog et al., 2006) [6] (Geske et al., 2005) [7] (Geske et al., 2008a) [8] (Kim et al., 2009a), and [9] (Kim et al., 2009b).

Several comprehensive SAR analyses of synthetic non-natural AHL analogs targeting *P. aeruginosa* have been reported. In 2008 Geske et al. published a review that summarizes SARs for non-natural AHL analogs in a range of bacterial species (Geske et al., 2008b), while Spring et al. (Galloway et al., 2011) in 2011 published a SAR analysis on AHL QS modulators. Below is a short summary of the SAR trends highlighted in these reviews, and Figure 14 outlines the most potent inhibitors. Geske et al. delineated some broad activity trends for the AHL mimics (see below). These trends are highlighted with colored spheres in Figure 14 for AHL analogs found to inhibit QS in *P. aeruginosa*. **Trend 1** concerns the length of the acyl chain, which in general have to be close to the length of the natural AHL to have high activity. **Trend 2** and **Trend 3** relate to the third carbon on the acyl chain, and in **Trend 2** it is seen that a carbonyl group in this position is important for activity. However, the carbonyl is not essential, and **Trend 3** summarizes AHL inhibitors that do not contain this carbonyl. **Trend 4** highlights the importance of stereochemistry where the L-stereoisomer of the natural AHL lactone ring is important. However, studies investigating racemic mixtures has also resulted in identification of good inhibitors. Therefore, it is uncertain whether the L-isomer is the only active species. **Trend 5** concerns modifications of the lactone ring, which has been shown to be tolerated in some systems but most often has led to a decrease in activity. Lastly, **Trend 6** shows AHL inhibitors that incorporate aromatic functionalities either by replacing the lactone ring or as substituent on the acyl chain.

**Figure 15:**
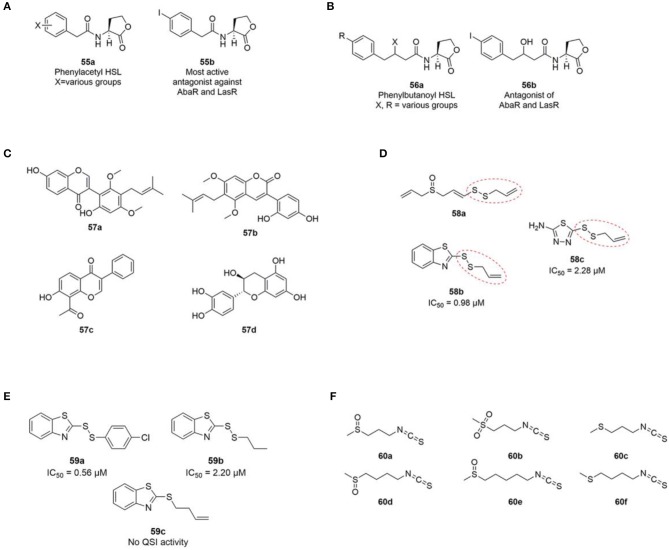
**(A)** General phenylacetyl HSL (PHL) (**55a**) and the most active PHL antagonist (**55b**) against AbaR (Stacy et al., 2012) and LasR (Geske et al., 2008c). **(B)** General phenylbutanoyl HSL (PBHL) (**56a**) and one of the most active PBHL antagonist (**56b**) against AbaR and LasR (Gerdt et al., 2017). **(C)** Structures of licoricone (**57a**), glycyrin (**57b**), glyzarin (**57c**) and flavan-3-ol catechin (**57d**). **(D)** Structure of ajoene (**58a**) and two additional inhibitory QSI inhibitors (**58b** and **58c**) identified from a random screening by Fong et al. (2017). **(E)** Ajoene analogs synthesized by Fong et al. (2017). **(F)** Structures of iberin (**60a**), cheirolin (**60b**), iberverin (**60c**), sulforaphane (**60d**), alyssin (**60e**) (Jakobsen et al., 2012a), and erucin (**60f**) (Ganin et al., 2013) with QS inhibitory activity against *P. aeruginosa*.

In 2012 Stacy et al. published a SAR study of non-natural AHL analogues' ability to activate and inhibit *A. baumannii* QS by agonizing or antagonizing the AbaR receptor (Stacy et al., 2012). In general, the strongest AbaR antagonist contained aromatic acyl groups. Phenylacetanoyls homoserine lactones (PHLs) (**55a**) with substituents on the phenyl ring showed good antagonistic activity. Among the halogenated PHLs, antagonistic activity was found to increase with increasing size and decreasing electronegativity of the halogen. For example, the iodo PHLs (**55b**) was strongly inhibitory as the most active group followed by bromo and chloro being good to moderate inhibitors and fluoro PHLs being weakly inhibitory (Figure 15). Compounds containing substitutions in the 3-position of the PHL aromatic ring were found to generally show stronger inhibition, whereas inhibitor activity was lowered when substitutions were introduced in the 2- and 4-positions. A previous study reported a similar trend with non-native AHLs containing a PHL group showing antagonistic activity of the *P. aeruginosa* LasR protein (Geske et al., 2008c). Stacy et al. suggested that the mode of action of the investigated class of PHLs in LasR and AbaR could be similar (Stacy et al., 2012).

Based on the activity of PHL described above, a recent study investigated two carbon extended *N*-(phenylbutanoyl) L-homoserine lactone (PBHL) ligands for inhibition of AbaR and LasR activity. The examined PBHL either contained unsubstituted or chlorine- or iodine-substituted phenyl groups and varied in the oxidation states of the 3-position (**56a**, Figure 15). It was reported that inhibition of LasR moderately depended on oxidation state, whereas AbaR inhibition was not significantly affected by the oxidation state of the PBHL ligand.

### Studies on Non-AHL-Based QS Modulators

The mode of actions of non-AHL like QS inhibitory compounds have in many cases not been specified questioning which part of the QS system is targeted. Therefore, is it possible that some of the compounds presented in the following section do not target the QS system through modulation of the LuxR-type receptor specifically. In addition, there is often a considerable difference between the molecular structures of the identified QS inhibitors from various studies of non-AHL based QS modulators which limit the number of compounds to build adequate SAR studies. Nevertheless, the structures of the different identified QS inhibitors are relevant for further discussion of what might be potentially active compounds.

Some of the first identified and intensively studied QS inhibitors originating from natural sources were the brominated furanones. Since the bioactivity of furanones, extracted from the marine algae *Delisea pulchra*, was hypothesized to exert its activity by paralyzing the AHL regulated QS system (Givskov et al., 1996), several studies have investigated QS modulation activity of a great number of furanone derivatives. In 2003, the Givskov group delivered proof of concept regarding QS inhibition as an antimicrobial principle (Hentzer et al., 2003). With chemically modified halogenated furanones they showed a significant impact on QS controlled gene expression (first transcriptomic analyses to be undertaken), as well as decreases in QS regulated virulence factors, and accelerated clearance of *P. aeruginosa* in a lung infection mouse model (Hentzer et al., 2003; Wu et al., 2004). For a more detailed description of investigated furanones together with related structural analogs the reader is referred to Galloway et al. (2011).

Phytochemicals such as flavonoids, flavanones, polyphenols, furacoumarins, and hydrolysable tannins has been shown to disrupt the QS system in a number of different bacteria (Kalia et al., 2013). By bioassay guided fractionation of *Glycyrrhiza glabra*, commonly known as liquorice, a methanol extract was shown to inhibit motility and biofilm formation of *A. baumannii*. In addition, the production of 3-OH-C12-HSL was significantly reduced indicating disruption of the QS system. Q-TOF MS and Q-TRAP MS/MS analysis of the extract suggested the presence of the following three flavonoids; licoricone (**57a**), and glycyrin (**57b**) and glyzarin (**57c**) (Figure 15) (Bhargava et al., 2015). The flavonoid flavan-3-ol catechin (**57d**) extracted from the bark of *Combrenum albiflorum* has been reported to decrease the expression of *P. aeruginosa* QS regulated genes as well as to inhibit the production of QS regulated virulence factors (Vandeputte et al., 2010).

A number of sulfur containing compounds has been reported as QS inhibitors of *P. aeruginosa*. Ajoene **58a** (Figure 15) from crushed garlic was reported to inhibit a subset of QS controlled genes including rhamnolipid production, and to increase the susceptibility of *P. aeruginosa* biofilms to tobramycin and significantly decrease *P. aeruginosa* infection in the lungs of mice (Jakobsen et al., 2012b). Ajoene displays its activity by lowering the expression of the two small RNAs RsmY and RsmZ located upstream of the central QS system in *P. aeruginosa* (Jakobsen et al., 2017b). From a random screening of a chemical compound library two sulfur containing compounds **58b** and **58c** (Figure 15) with structural similarities to ajoene were reported as QS inhibitors of *P. aeruginosa* (Fong et al., 2017). Both compounds share the same allylic disulphide bond with ajoene marked with a red dotted line in Figure 15.

Subsequently, syntheses of 25 disulphide bond-containing analogs were carried out to investigate the importance of the allyl disulphide group for bioactivity (Fong et al., 2017). The authors reported that the allyl group was not strictly important for QS activity, which was shown with the saturated analog **58b** that displayed excellent QS inhibitory activity (Figure 15). A *p*-chlorophenyl derivative (**59a**) showed the highest QSI activity among the tested compounds, whereas the absence of disulphide bond diminished the QS inhibitory activity completely as shown with **59c** in Figure 15. In addition, two compounds devoid of the benzothiazole functional group did not display any QS inhibitory activity. The two compounds **59b** and **59a** were reported to decrease production of the three important QS regulated virulence factors; elastase, pyocyanine, and rhamnolipid. In addition, both compounds significantly decreased *P. aeruginosa* bacterial counts in a foreign-body implant mouse model compared to an untreated control group. This relative small SAR analysis of ajoene analogs indicates that the presence of a disulphide bond is important for bioactivity. In addition, the presence of a benzothiazole moiety increases the QS inhibitory activity substantially compared with ajoene.

Additional sulfur containing compounds have been identified as QS inhibitors of *P. aeruginosa*. By means of bioassay-guided fractionation of horseradish, 1-isothiocyanato-3-(methylsulfinyl) propane, commonly known as iberin (**60a**, Figure 15) was identified as an efficient QS inhibitor of *P. aeruginosa* (Jakobsen et al., 2012a). Iberin **60a** is a natural isothiocyanate with a sulfinyl group found in many members of the Brassicaceae family. Together with iberin **60a**, the following four related structural isothiocyanate analogs was reported as inhibitors: cheirolin (**60b**), iberverin (**60c**), sulforaphane (**60d**) and alyssin (**60e**) (Figure 15), however, they all showed lower QS inhibitory activities than iberin **60a** (IC50 values of ~2, 6, 10, and 30 times higher, respectively). Another study also reported sulforaphane to inhibit the QS system of *P. aeruginosa* together with the isothiocyanate erucin (**60f**, Figure 15) extracted from broccoli (Ganin et al., 2013).

Taken together, the number of different sulfur containing compounds that have been reported with excellent QS inhibitory activities makes it an interesting class of molecules to be studied further. The identified compounds have little structural similarity to native AHLs. This can be an advantage compared to AHL like compounds when considering that the homoserine lactone moiety is unstable at alkaline pH and potentially can be degraded by mammalian lactonases.

## Conclusions and Perspectives

According to WHO and UN, antimicrobial resistance is rising to unmanageable levels in all parts of the world and has become a top global threat. This grim perspective is paralleled by the fact that only two new classes of antibiotics have been clinically approved over the past 30 years. So far, the majority of clinically approved antibiotics have been designed to efficiently kill free-living (planktonic), growing bacteria. In the environment however, the preferred life-mode of bacteria is as densely packed microcolonies concealed in a protective matrix of biopolymers that offer protection against the otherwise lethal action of a wide variety of environmental stressors including antimicrobials. In this biofilm life-mode, bacteria are also believed to cause a significant number of human microbial infections. Nosocomial infections are estimated to be the fourth leading cause of death in the U.S. with 2 million cases annually, and about 60–70% of these nosocomial infections are caused by bacterial biofilms (Bryers, 2008). Because of the general neglect of the biofilm-mode in past antibiotic development, the central problem with biofilm infections is that the involved bacteria are generally not susceptible to our present assortment of antibiotics. Adding to this the resistance to the antimicrobial activities of the immune system in general, the biofilm mode offers an almost unlimited capacity to survive in the infected host (Costerton et al., 1999; Rybtke et al., 2015).

With the increase in infectious diseases showing multiple resistances and the paralleled downsizing in antibiotic development there is, more than ever, an urgent need for development of antimicrobials with new and different modes of action. Such antimicrobials may be designed to include non-lethal targets associated with the biofilm life-mode, where the chemistry and mode of actions of some of those compounds have been reviewed in the present paper. Conceptually such drugs function to attenuate biofilm formation and/or pathogenicity without obstructing processes that are essential for bacterial growth and reproduction. Among the few identified targets are two key biofilm gateways; the one that controls the switch between the biofilm-planktonic life modes in responses to the internal levels of c-di-GMP, and QS which governs the production of virulence factors and some of the protective mechanisms operating in the biofilm mode. Proof of concept driven research initiated more than 20 years ago by us have shown the signaling gateways to be viable drug targets for a new class of what can generally be referred to as non-lethal antibiotics (Hentzer et al., 2003). It has been postulated, but not directly demonstrated, that such modes of drug action would reduce the selection pressure for resistant bacterial variants. While this may to a certain extent be correct for QS, we have unpublished experimental data showing that in an environment where biofilm formation promotes growth and provides a hold-fast of bacteria, chemical obstruction of biofilm formation raises a significant selection pressure for insensitive mutants and resistance seems to arise as frequently as it does to the action of conventional antibiotics.

We and others are currently in pursuit of the development of chemical compounds capable of preventing formation of biofilms. Most of the work done so far aims at targeting (inhibiting) c-di-GMP synthesis. One of the rationales for this is the highly conserved composition of the GGDEF domains in multiple Gram negative species. Aiming for this target would then be expected to result in development of broad spectrum anti-biofilm drugs. We have been following this strategy too, but with disappointing results. We are currently pursuing stimulation of PDE activities to accomplice a reduction in the cellular c-di-GMP content. This strategy comes from our findings that overexpression of an *E. coli* PDE (PdeH) in *P. aeruginosa* plummets the c-di-GMP level and results in massive biofilm dispersal (Christensen et al., 2013). This concept is functional *in vivo* as judged from our mouse implant model of infection (Christensen et al., 2013). However, success of this strategy depends on selection of the right PDE, since overexpression of some of the PDEs in *P. aeruginosa* evidently do not lead to biofilm dispersal (Chambers et al., 2017), which is in agreement with the emerging theme that DGCs, PDEs and c-di-GMP effectors often work via protein-protein interactions. Treatment of biofilm infections by means of large scale dispersal of bacteria from the biofilms, would require combinatorial treatments with conventional antibiotics that eradicate the dispersed bacteria.

Whereas, the success criteria for lethal antibiotics have largely been based on low MIC values, the process of designing non-lethal antibiotics that would in turn work in concert with their lethal counter parts is significantly more elaborative and challenging. As emphasized in this review, small molecules continue to emerge as efficient modulators of bacterial biofilm formation. An increasing number of new molecular scaffolds and chemotypes, derived from both natural products and of entirely synthetic origin, have been developed. The reported compounds are currently useful as tools to study the molecular mechanisms involved in biofilm formation, growth and dispersal, but may also serve as hits to guide early discovery efforts. There is academic and industrial consensus on the unique promise for translational applications, such as new antimicrobial medicines, anti-biofouling and crop protection agents, but the path forward for small-molecule biofilm modulation remains long. Driven by academic research efforts, it is only natural that most SAR studies have been balancing synthetic feasibility and *in vitro* studies as a prequel to devoted medicinal chemistry campaigns. For example, virtually no knowledge is available on the performance of biofilm modulating scaffolds in integrated ADME/TOX evaluation studies, pointing to all the usual challenges of small molecule drug discovery. As a further complication, a combination of poor assay standardization and studies on clinically irrelevant bacterial strains has significantly hampered target evaluation and drugability assessment. The biofilm scientific community must address the myriad of hard-to-reproduce biological data to leverage the value of the many novel compounds emerging from academic laboratories, and ultimately secure the fundamental basis of clinical translation.

## Author Contributions

All authors listed have made a substantial, direct and intellectual contribution to the work, and approved it for publication.

### Conflict of Interest

The authors declare that the research was conducted in the absence of any commercial or financial relationships that could be construed as a potential conflict of interest.
